# A Targetable N-Terminal Motif Orchestrates α-Synuclein Oligomer-to-Fibril Conversion

**DOI:** 10.1021/jacs.4c02262

**Published:** 2024-04-29

**Authors:** Jaime Santos, Jorge Cuellar, Irantzu Pallarès, Emily J. Byrd, Alons Lends, Fernando Moro, Muhammed Bilal Abdul-Shukkoor, Jordi Pujols, Lorea Velasco-Carneros, Frank Sobott, Daniel E. Otzen, Antonio N. Calabrese, Arturo Muga, Jan Skov Pedersen, Antoine Loquet, Jose María Valpuesta, Sheena E. Radford, Salvador Ventura

**Affiliations:** † Institut de Biotecnologia i Biomedicina and Departament de Bioquímica i Biologia Molecular, Universitat Autònoma de Barcelona, Bellaterra, Barcelona 08193, Spain; ‡ Department of Macromolecular Structures, Centro Nacional de Biotecnología (CNB-CSIC), Madrid 28049, Spain; § Astbury Centre for Structural Molecular Biology, School of Molecular and Cellular Biology, Faculty of Biological Sciences, University of Leeds, Leeds LS2 9JT, U.K.; ∥ Univ. Bordeaux, CNRS, Bordeaux INP, CBMN, UMR 5248, IECB, Pessac 33600, France; ⊥ Instituto Biofisika (UPV/EHU, CSIC) y Dpto. de Bioquímica y Biología Molecular, Facultad de Ciencia y Tecnología, Universidad del País Vasco, Barrio Sarriena S/N, Leioa 48940, Spain; # Interdisciplinary Nanoscience Center (iNANO) and Department of Molecular Biology and Genetics, Aarhus University, Gustav Wieds Vej 14, Aarhus C 8000, Denmark; ¶ Interdisciplinary Nanoscience Center (iNANO) and Department of Chemistry, Aarhus University, Gustav Wieds Vej 14, Aarhus C 8000, Denmark

## Abstract

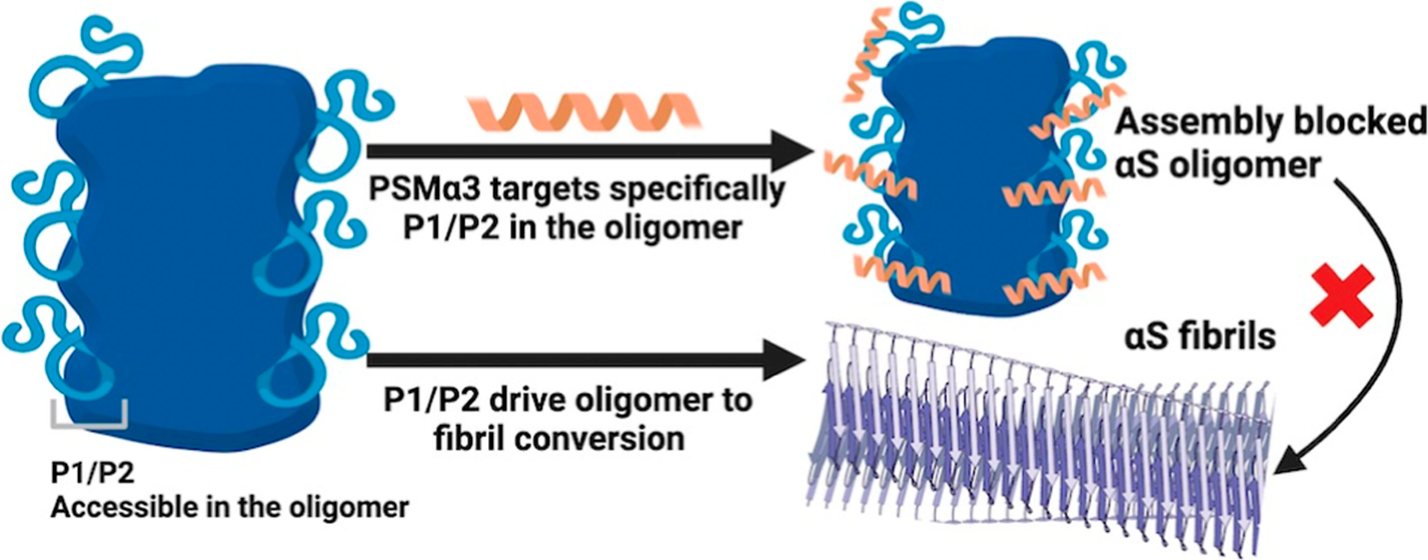

Oligomeric species populated during α-synuclein aggregation are considered key drivers of neurodegeneration in Parkinson’s disease. However, the development of oligomer-targeting therapeutics is constrained by our limited knowledge of their structure and the molecular determinants driving their conversion to fibrils. Phenol-soluble modulin α3 (PSMα3) is a nanomolar peptide binder of α-synuclein oligomers that inhibits aggregation by blocking oligomer-to-fibril conversion. Here, we investigate the binding of PSMα3 to α-synuclein oligomers to discover the mechanistic basis of this protective activity. We find that PSMα3 selectively targets an α-synuclein N-terminal motif (residues 36–61) that populates a distinct conformation in the mono- and oligomeric states. This α-synuclein region plays a pivotal role in oligomer-to-fibril conversion as its absence renders the central NAC domain insufficient to prompt this structural transition. The hereditary mutation G51D, associated with early onset Parkinson’s disease, causes a conformational fluctuation in this region, leading to delayed oligomer-to-fibril conversion and an accumulation of oligomers that are resistant to remodeling by molecular chaperones. Overall, our findings unveil a new targetable region in α-synuclein oligomers, advance our comprehension of oligomer-to-amyloid fibril conversion, and reveal a new facet of α-synuclein pathogenic mutations.

## Introduction

The aggregation of α-synuclein (αS), a 140-residue intrinsically disordered protein, is a defining hallmark of Parkinson’s disease and related synucleinopathies.^1−3^ In these disorders, αS self-assembles into amyloid fibrils that accumulate in the brain of patients, forming insoluble deposits known as Lewy bodies and Lewy neurites. The aggregation landscape of αS is dynamic, involving the formation of transient oligomeric species that precede and coexist with the final amyloid fibrils.^4−9^ αS oligomers are nonfibrillar soluble species that act as key kinetic intermediates in amyloid formation^6^ and contribute to gain-of-toxic interactions and disruption of cellular processes.^10,11^ Therefore, αS oligomers emerge as promising targets for therapeutic and diagnostic interventions,^12^ particularly during the early stages of the disease.

Over the past decade, there has been a growing interest in unraveling the structure, formation, and conversion to fibrils of αS oligomers, taking advantage of the ability to kinetically trap these species.^8,13−15^ Yet, their highly dynamic nature^14^ poses a technical limit for structural investigations, ultimately hampering the advancement of oligomer-targeting therapies. This emphasizes the need for alternative strategies to investigate the conformational and kinetic properties of αS oligomers.

**Figure 1:**
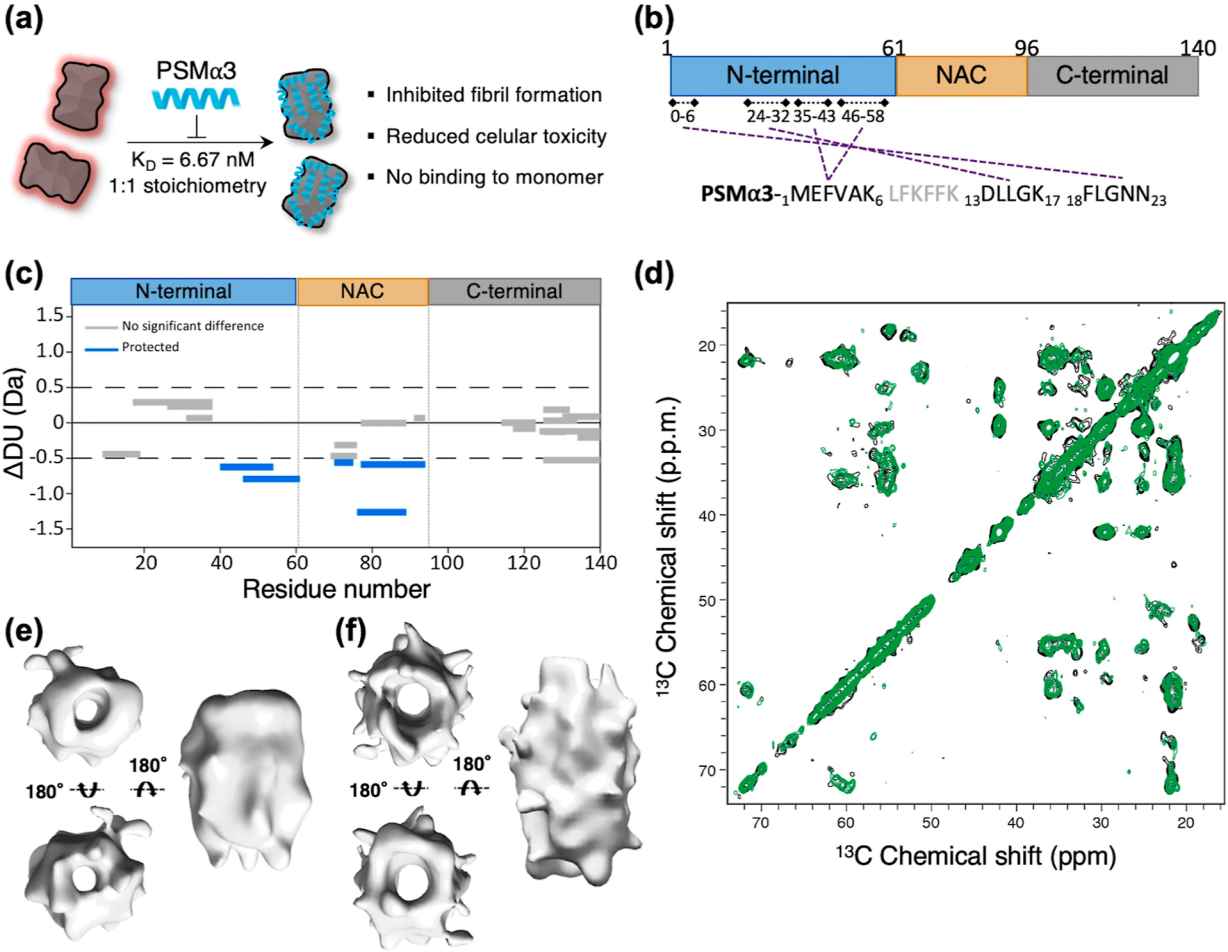
PSMα3 binding to αS oligomers. (a) Schematic representation of PSMα3 binding and activities. (b) Cross-linking map representing PSMα3 contacts with αS oligomers. (c) Wood’s plots showing the difference in deuterium uptake (ΔDU) when comparing αS oligomers in the complex with PSMα3 and free αS oligomers by HDX-MS at the 60 s exposure time point. Peptides colored blue are protected from exchange in the presence of PSMα3 (see the Experimental Section), suggesting that they are less solvent-exposed and/or participate in more inter/intraprotein hydrogen bonding in the presence of PSMα3. (d) 2D 13C–13C PDSD correlation spectra (mixing time of 50 ms) of oligomers (black) and oligomers + PSMα3 (green). (e) 3D reconstruction of αS oligomers in the absence of PSMα3 (18.5 Å resolution). (f) 3D reconstruction of αS oligomers in the complex with PSMα3 (19 Å resolution).

Phenol-soluble modulin α3 (PSMα3) is a 22-residue amphipathic α-helical peptide that binds αS oligomers with low nanomolar affinity and a 1:1 (αS/PSMα3) stoichiometry.^16^ The tight binding of PSMα3 to oligomers contrasts with the lack of any detectable interaction with monomeric αS, underscoring the existence of an oligomer-specific binding site for this peptide (Figure 1a). PSMα3 binding abrogates oligomer-associated neurotoxicity and inhibits αS aggregation by blocking oligomer-to-fibril conversion,^16^ thereby interfering with molecular events crucial for pathogenesis. These findings suggest that binding of PSMα3 to αS oligomers may be mediated by a therapeutically relevant, oligomer-specific motif.

Driven by this idea, we characterized the binding of PSMα3 to αS oligomers to identify a new oligomer-specific region implicated in αS pathogenesis. By combining an array of structural, biophysical, and biochemical approaches, we found that PSMα3 interacts primarily with a discrete binding site within the N-terminal region of αS, encompassing residues 36–61, which overlap with two regions (P1 and P2) reported to be “master controllers” of αS aggregation.^17−20^ We characterized the roles of P1 and P2 in the context of αS oligomers and found that these regions are critical for the oligomer-to-fibril transition. This structural conversion process is tightly regulated by the sequence of this N-terminal region of αS. Accordingly, we show that a familial mutation within this region associated with early onset Parkinson’s (G51D) causes a local conformational fluctuation that delays oligomer-to-fibril conversion, resulting in the accumulation of oligomers that resist disaggregation by molecular chaperones.

Overall, we here identify a disease-relevant αS region fundamental to oligomer-to-fibril conversion. This sequence defines an oligomer-specific motif that can be targeted by molecular ligands, revealing uncharted territory for the design of oligomer-directed therapeutic and diagnostic tools.

## Results

### PSMα3 Binds to a Defined Motif in the N-Terminal of αS Oligomers

To identify the PSMα3 binding site in αS oligomers, we first investigated αS-PSMα3 interactions using cross-linking mass spectrometry (XL-MS) and the zero-length cross-linker, 4-(4,6-dimethoxy-1,3,5-triazin-2-yl)-4-methyl-morpholinium chloride (DMTMM). Isolated oligomers were prepared as previously described,^13^ incubating 800 μM of monomeric αS for 20 h at 37 °C quiescently, followed by centrifugation-based fractionation. Oligomer-PSMα3 complexes were generated by incubating oligomers with a 3-fold molar excess of PSMα3 and subsequently removing the unbound peptide by centrifugal filtration. DMTMM cross-linking of the oligomer-PSMα3 complexes revealed four different contact sites between PSMα3 and the N-terminal domain of αS, encompassing residues 1–6, 24–32, 35–43, and 46–58 (Figure 1b).

We next sought to confirm the principal segments defining the PSMα3-oligomer interface using hydrogen–deuterium exchange mass spectrometry (HDX-MS). Solvent-exposed residues lacking protein–protein hydrogen bonds incorporate deuterium more rapidly than buried residues or those engaged in inter/intramolecular hydrogen bonding. Thus, αS amino acids contributing to the PSMα3 binding site should exhibit lower deuterium uptake when PSMα3 is bound. We used differential HDX-MS to compare the extent of deuterium incorporation in αS oligomers in the absence and presence of PSMα3. In the presence of PSMα3, two αS peptides covering residues 40 to 61 showed significant protection from deuterium uptake (Figures 1c and S1). Together with the XL-MS contacts, this suggests that this N-terminal region constitutes the primary PSMα3 binding site within αS oligomers. Additionally, significant protection from deuterium uptake was identified in three peptides in the NAC domain. Considering the lack of cross-links in this segment, this observation suggests that PSMα3 binding induces a conformational rearrangement of the NAC region, which causes a change in solvent accessibility and/or hydrogen bonding in this region.

To address this question, we also investigated the interaction between PSMα3 and αS oligomers using magic-angle spinning solid-state nuclear magnetic resonance (MAS-ssNMR). MAS-ssNMR has been used previously to define the rigid core of these αS oligomers, comprising residues 70 to 88 within the NAC region.^14^ More mobile segments were assigned, spanning residues 1–20 and 90–140.^14^ Thus, even if the primary PSMα3 binding site (region 35–61, as determined by XL-MS/HDX-MS, Figure 1b,c) cannot be assessed by ssNMR, this technique provides a means to detect conformational shifts within the oligomer’s rigid core upon peptide binding, validating our observations from HDX-MS (Figure 1c). We recorded cross-polarization (CP) and insensitive nuclei enhanced by polarization transfer (INEPT) experiments to measure the ^13^C signals in rigid and mobile segments of αS oligomers, respectively, in the absence or presence of PSMα3. The chemical shifts in the CP-based 2D spectra were identical in both cases (Figure 1d). This unique set of resonances was assigned as residues 70 to 89 based on previous studies of αS fibrils (Table S1 and Figure S2a)^21^ and in agreement with those documented for a previously characterized toxic αS oligomer by ssNMR.^14^ Given that no chemical shift differences were observed in this region in the presence of PSMα3, this suggests that the protection in this region from deuterium uptake in the presence of PSMα3 detected by HDX-MS is not a result of direct binding or a major structural reorganization of the core. Similarly, only minor chemical shift differences were detected in the INEPT-based 2D spectra (reporting on mobile αS residues 1–20 and 90–140) (Figure S2b), consistent with the principal PSMα3 binding site encompassing residues 40 to 61, which are in an intermediate motional regime not accessible to CP and INEPT. Despite the absence of significant chemical shift differences, we noted a clear increase in the CP signal and CP/INEPT ratio in the presence of PSMα3 while sharing the same water hydration dynamics (Figure S2c–e). This implies a rigidification or loss of dynamic excursions of the oligomer’s ssNMR-detectable residues upon peptide binding. Considering this, it is conceivable that the protection from deuterium exchange observed in the NAC region of oligomeric αS in the presence of PSMα3 stems from a binding-induced increase in the rigidity.

Consistent with the ssNMR data, PSMα3 binding reduced the conformational heterogeneity of αS oligomers, relative to the oligomers alone, as judged by negative stain electron microscopy (nsEM) images (Figure S3a,b). This was further sustained by cryo-electron microscopy (cryoEM) 3D density reconstruction of the two sets of particles, revealing a cylindrical architecture with a central hollow core (Figures 1e,f and S3c–e), consistent with previous observations of αS oligomers.^13^ While the overall architecture of the αS oligomers remained unaltered upon PSMα3 binding, the associated rigidification effect promoted an increase in the structural order in the PSMα3-oligomer complexes, noticeable in the 2D classes generated during the 3D reconstruction (Figure S3c–e). End-on views showed a 6-fold symmetry, also visible in the 3D reconstruction of oligomers, both in the absence and presence of PSMα3.

Overall, our data indicate that PSMα3 binds to a specific site within the N-terminal domain of αS, spanning residues 36 to 61. In addition, PSMα3 binding rigidifies but does not cause a significant structural reconfiguration in the oligomer rigid core.

### PSMα3 Binding Site in αS Oligomers Is Partially Collapsed and Solvent Accessible

**Figure 2:**
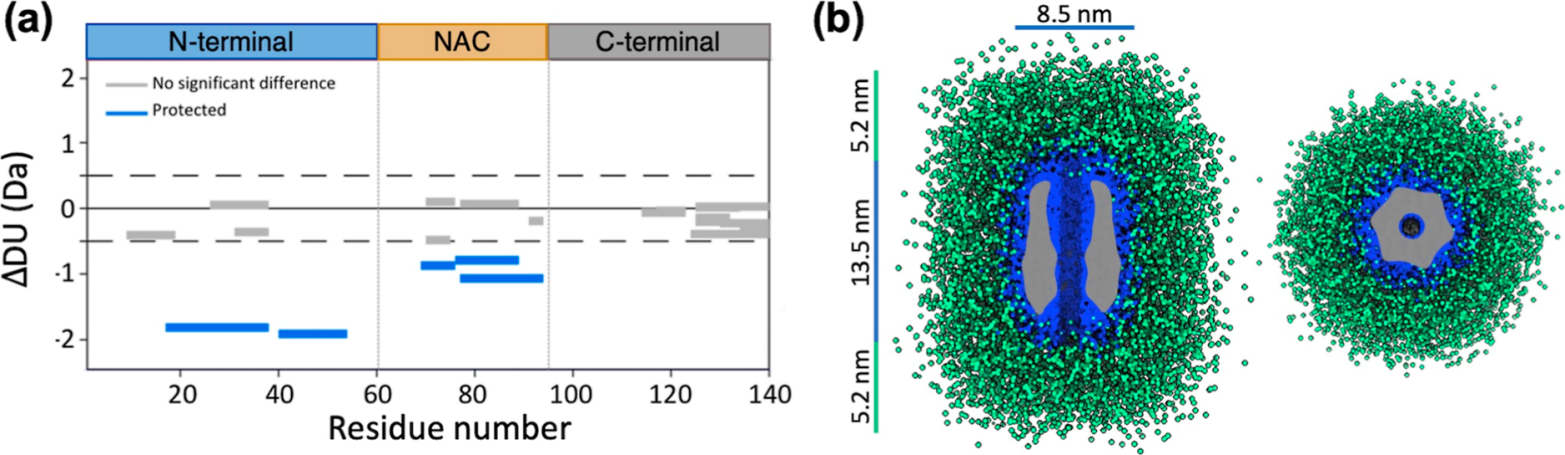
Dynamics of the N-terminal region of αS in the oligomer. (a) Wood’s plots showing the difference in deuterium uptake (ΔDU) between αS monomers and oligomers by HDX-MS at the 60 s exposure time point to deuterium. Peptides colored blue are significantly protected from exchange in αS oligomers compared with monomeric αS. (b) Two views of the SAXS-based 3D reconstruction of αS oligomers. The compact core (blue) is surrounded by an outer disordered shell (green). The cryoEM density map is shown inside the oligomer core (gray).

To gain further insights into the conformation and dynamic properties of the different regions in oligomeric αS, we analyzed the differential deuterium uptake between αS monomers and oligomers using HDX-MS. Peptides in both the N-terminal region and NAC domain become protected upon oligomer formation (Figures 2a and S1), whereas no differences in deuterium incorporation in the C-terminal region were observed when comparing αS monomers and oligomers, suggesting that this region remains flexible and disordered in the assembled state. The enhanced protection in the NAC domain upon oligomer formation was expected, considering that this region forms the rigid and structured core of the oligomers, according to the ssNMR CP data. Remarkably, the degree of protection was greater for peptides in the N-terminal region compared to those in the NAC domain, suggesting that this N-terminal segment undergoes significant structural remodeling from the initially disordered state of the monomer. We next applied XL-MS using DMTMM to identify αS–αS contacts within the oligomer. These results confirmed that the enhanced protection from deuterium exchange in the N-terminal domain coincides with the formation of contacts both between different residues within this domain (intradomain), as well as interdomain interactions between N-terminal and NAC regions (Figure S4).

Finally, we used small-angle X-ray scattering (SAXS) to probe the conformational properties of the dynamic and disordered αS regions in the oligomer (Figures 2b and S5a and Table S2). The oligomer compact core (blue) was modeled as a superellipsoid with a central cylindrical hole and showed dimensions (Table S2) that align with the cryoEM visible map (gray). This central region is surrounded by an outer shell of disordered tails (green), resulting in an oligomer radius of gyration of 76.1 ± 0.3 Å and dimensions consistent with previous reports (Figure 2b).^8,22^ Notably, our experimental SAXS data fitted better to a model with a single random coil chain per monomer rather than two (Figure S5b), as would be anticipated if both the N- and C-terminal domains remained fully disordered. In this way, the disordered fuzzy coat was modeled to encompass 48% of the αS residues (Table S2), whereas the complete N- and C-terminal αS domains account for 76% of the αS sequence. In contrast, the principal contributors to the oligomer INEPT spectra^14^ (residues 1–20 and 90–140) account for 50% of the αS sequence.

Together, these results indicate that the primary constituents of the outer disordered corona of the oligomers are the C-termini, with a contribution from the 20 N-terminal residues of αS. Other residues in the N-terminal domain, containing the PSMα3 binding site, presumably form a partially structured or collapsed conformation that is sufficiently dynamic to be undetectable in the ssNMR CP spectra. The high affinity of PSMα3 for this region and the 1:1 (αS/PSMα3) stoichiometry underscore the specificity and accessibility of these N-terminal segments for interactions within the oligomer assembly.

### N-Terminal P1 and P2 Regions of αS Control Oligomer-to-Fibril Conversion

The PSMα3 binding site in the αS oligomers encompasses two regions in the N-terminal of αS known to be pivotal for amyloid formation: P1 (36–42) and P2 (45–57).^17,18^ These sequences act as “master controllers” of αS amyloid formation in vitro and in *Caenorhabditis elegans*, with deletions or point mutations in P1 and P2 suppressing or delaying amyloid formation.^17,18^ Since binding of PSMα3 blocks oligomer-to-fibril transition,^16^ we investigated the specific contributions of P1 and P2 to this conformational conversion.

**Figure 3:**
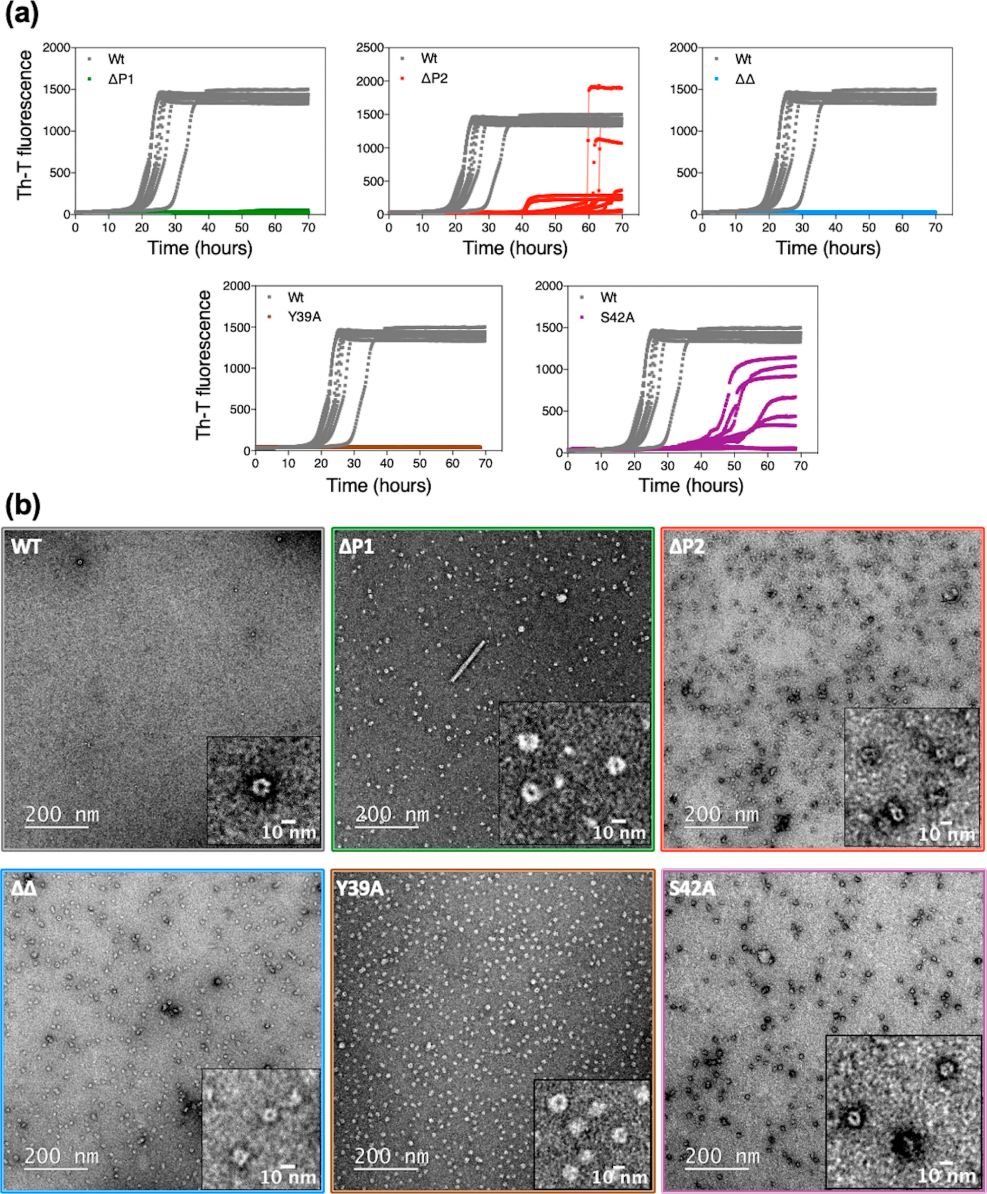
Contribution of PSMα3 binding site to oligomer-to-fibril conversion. (a) Kinetics of amyloid formation of the WT, ΔP1, ΔP2, ΔΔ, Y39A, and S42A variants monitored using Th-T fluorescence. (b) Representative nsEM images of the oligomeric fraction of the WT (top left), ΔP1 (top middle), ΔP2 (top right), ΔΔ (bottom left), Y39A (bottom middle), and S42A (bottom right) isolated at the end point (WT, ΔP1, ΔP2, ΔΔ, and Y39A) or after 28 h of assembly (S42A).

We assayed the impact of P1 and P2 deletions (ΔP1 and ΔP2) on αS amyloid formation using thioflavin-T (Th-T) fluorescence as a reporter. In agreement with previous results, the deletion of P1 (ΔP1) inhibited αS amyloid formation, whereas the deletion of P2 (ΔP2) retarded aggregation under the conditions used^17,18^ (Figure 3a). nsEM of the end point of the aggregation reaction confirmed that wild-type (WT) αS formed mature amyloid fibrils. In contrast, none or a few fibrils were present in the ΔP1 and ΔP2 variants at the end point of the reactions (Figure S6). We then applied a centrifugation-based protocol to study the presence of oligomeric species in those samples (see the Experimental Section). As expected, few low-molecular-weight species (nonsedimentable particles with a molecular weight >100 kDa) were visible at the end point of the WT αS amyloid assembly reaction (Figure 3b). In contrast, the primary components in the low-molecular-weight fraction for ΔP1- and ΔP2-inhibited reactions were oligomers identical in shape and size to WT oligomers (Figure 3b). Similar results were obtained when both P1 and P2 were deleted in tandem (ΔΔ) (Figures 3a,b and S7). To further validate that P1 and P2 are not required for oligomer formation, we verified that the ΔP1, ΔP2, and ΔΔvariants can form kinetically trapped oligomers with the same morphology as that of WT αS (Figure S8). These findings indicate that while P1 and P2 are not necessary for αS oligomerization, they actively contribute to the conversion of oligomers into fibrils.

The modulation of amyloid formation by the P1 region has been shown to be dependent on specific residues.^18^ Hence, we characterized two αS variants, Y39A and S42A, which were shown previously to mimic the ΔP1 variant, inhibiting amyloid fibril formation.^18^ Consistent with prior results, the Y39A and S42A amino acid substitutions within the P1 region inhibited αS amyloid formation to different extents (Figures 3a and S7). In both cases, oligomers akin to those observed above for WT αS were the predominant species in the oligomeric fraction at the time points of maximal inhibition (the end point for ΔP1 and 28 h for ΔP2), indicating an impaired transition of oligomers to fibrils (Figure 3b). Notably, the WT αS and S42A proteins differ by just a single hydroxymethyl group, evidencing the precise control that subtle sequence changes can exert on the conversion of oligomers to fibrils.

To determine whether oligomer accumulation is specific to sequence changes or deletions in the P1 and P2 regions, we characterized an N-terminal truncated variant (ΔN11), which has been shown to result in delayed amyloid assembly due to decreased secondary nucleation.^7,23^ As expected, deletion of the N-terminal 11 residues inhibited amyloid formation, but oligomers were barely detectable in the low-molecular-weight fraction (Figures 3a and S9), consistent with the inhibition mechanism being distinct from the impaired oligomer-to-fibril conversion observed for ΔP1 and ΔP2. Furthermore, we confirmed that these first 11 N-terminal residues are dispensable for oligomerization as ΔN11 αS effectively assembles into WT-like oligomers under conditions used to generate the kinetically trapped WT αS described above (Figure S9).

Overall, these data demonstrate the essential role of P1 and P2 in facilitating oligomer-to-fibril conversion and define the mechanistic foundation for PSMα3 inhibition of αS aggregation. Based on this knowledge, we suggest that molecules that are able to bind P1 and P2 in the oligomeric state could possess intrinsic antiamyloidogenic properties by suppressing the oligomer-to-fibril transition.

### Familial G51D Mutation Impairs N-Terminal-Mediated Oligomer-to-Fibril Conversion and Chaperone-Assisted Disaggregation

**Figure 4:**
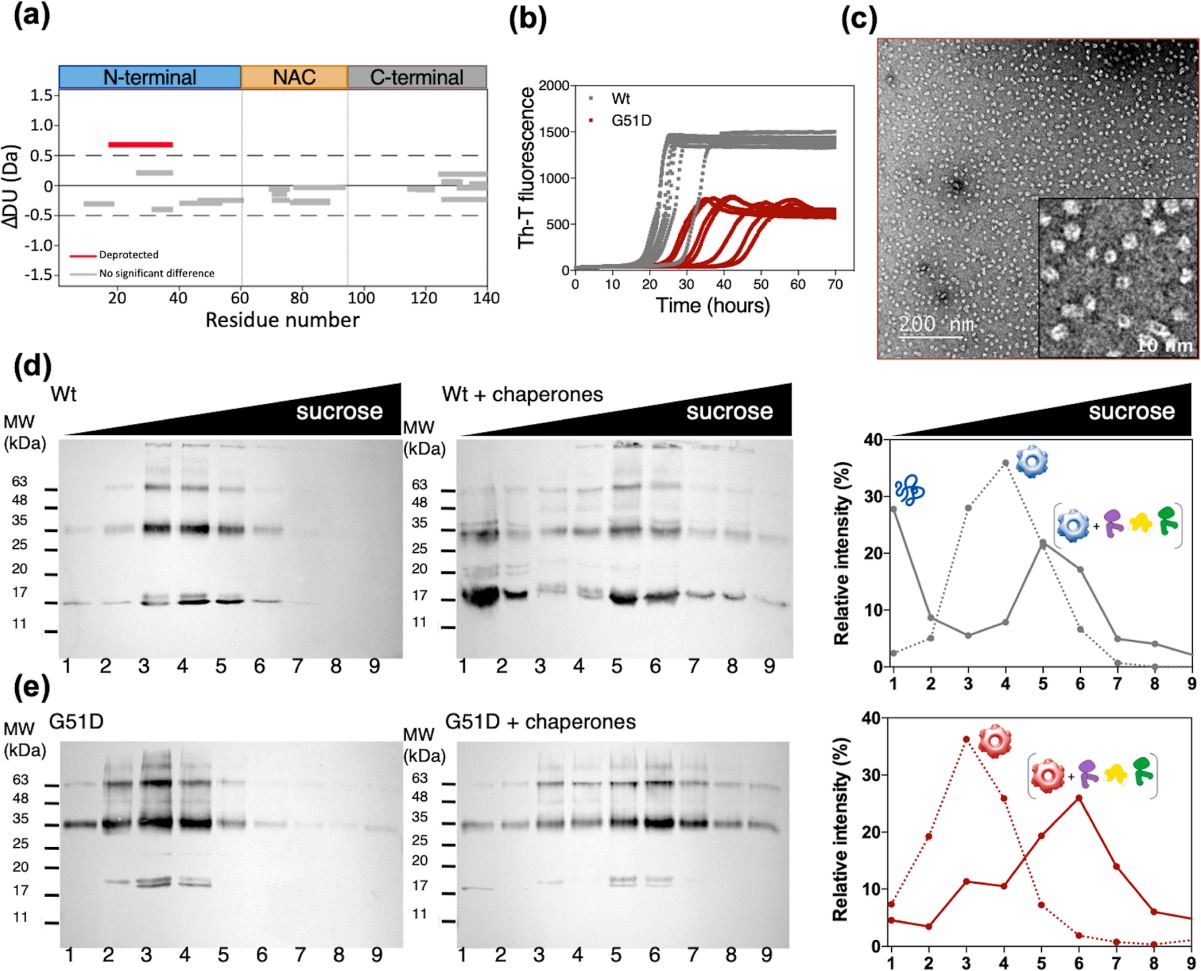
Effect of the familial G51D mutation on αS amyloid and oligomer formation and disaggregation by molecular chaperones. (a) Wood’s plot showing the relative solvent exposure/hydrogen bonding of G51D αS oligomers compared with that of WT αS oligomers by HDX-MS at the 60 s time point of exposure to deuterium. Deprotection from deuterium uptake occurs in the N-terminal region, as indicated by the peptide region colored red. (b) Assembly kinetics of G51D into amyloid fibrils monitored using ThT fluorescence. (c) Representative nsEM micrographs of the G51D oligomeric fraction after 28 h of assembly. (d,e) Sucrose-gradient fractionation of WT (d) and G51D (e) oligomers in the absence (left panels) or upon 2.5 h of incubation at 30 °C with the human disaggregase at αS/Hsc70 1:1.5 molar ratios (central panels). The distribution across the gradient was followed by Western blot analysis using an anti-αS antibody. The relative intensity of the immunoreactive bands in the Western blots was quantified for nontreated (dotted line) and treated (solid line) oligomers to illustrate their differential distribution (right panels).

Familial mutations in the gene encoding αS often lead to a more aggressive form of Parkinson’s disease.^2^ For instance, patients carrying the αS G51D mutation experience a more severe clinical course of the disease, characterized by earlier symptoms onset and significant psychiatric and autonomic dysfunctions.^24,25^ The G51D mutation localizes to the P2 region, with our findings suggesting that it may influence the conformational dynamics of the N-terminal domain and hence the ability of oligomers to convert into amyloid fibrils. This aligns with the previous observation that this amino acid change alters the oligomer conformation and induces a distinctive α-helical secondary structure component in their circular dichroism spectra,^26^ which we corroborated here (Figure S10). To delve deeper into the impact of this mutation on the properties of the oligomers, we analyzed the differential deuterium uptake of the WT and G51D αS oligomers. Compared with WT αS oligomers, G51D αS oligomers showed a significant increase in deuterium uptake in their N-terminal region involving residues 17–38, as exemplified by deprotection, impacting P1 (Figures 4a and S1). This increased deuterium accessibility indicates a conformational transition N-terminal to the mutation site, implying a long-range effect in the oligomer structure or dynamics exerted by the G51D amino acid substitution.

The hereditary G51D mutation is also known to attenuate αS aggregation,^27^ suggesting that the induced conformational rearrangement may also affect oligomer-to-fibril conversion. As observed for the synthetic mutants in P1 and P2, the delayed aggregation of G51D αS is associated with the accumulation of WT-like oligomers at the time point of maximal difference (*t* = 28 h) compared with the WT fibril formation kinetics (Figure 4b,c). The G51D variant thus exemplifies how a disease-associated mutation in the P2 region elicits a structural rearrangement of the N-terminal region of the αS oligomer, including the P1 sequence, which further impacts oligomer-to-fibril conversion.

The N-terminal region affected by the G51D substitution identified above encompasses a well-established Hsc70 binding site (residues 37–43).^28,29^ Hsc70 is a fundamental element of the mammalian chaperone disaggregation machinery, working synergistically with its cochaperones DNAJB1 and Apg2.^29,30^ We decided to assess whether the observed conformational differences in the Hsc70 binding site could alter its ability to be processed by chaperones. We hence monitored chaperone-mediated disaggregation of oligomers formed by WT and G51D αS using density-gradient centrifugation (Figures 4d,e and S11). WT αS oligomers were efficiently disaggregated into monomers that floated to the top of the sucrose gradient at αS/Hsc70 1:1.5 molar ratios, while G51D oligomers were barely solubilized under the same conditions, exhibiting a greater resistance to Hsc70-mediated disaggregation (Figure 4d,e). Sample fractionation also revealed that the remaining oligomers of both proteins bound DNAJB1 and Hsc70, moving to more dense fractions of the sucrose gradient (Figures 4d,e and S11). G51D oligomers are thus able to recruit the disaggregation machinery, but they are not effectively processed, leading to an unproductive interaction where the G51D αS oligomers kidnap these essential protein quality control elements.

## Discussion

The recent resolution revolution in cryoEM has significantly advanced our structural understanding of the fibrillar amyloid state.^31−33^ In contrast, the structure of intermediate oligomeric species remains largely uncharted,^34^ hampering the development of oligomer-focused therapeutic strategies, despite their potential clinical relevance. For instance, lecanemab, an FDA-approved monoclonal antibody, mitigates cognitive decline in Alzheimer’s disease by binding soluble Aβ aggregates (oligomers and protofibrils) with high selectivity over monomers.^35^

In this study, we characterized the binding site of PSMα3 within αS oligomers, leveraging this information to interrogate the oligomer structural properties and the molecular determinants of oligomer progression to the amyloid state. We demonstrate that a specific motif involving residues 36–60 in the N-terminal region of αS mediates PSMα3 selective binding to αS oligomers. This binding site has a distinct conformation in αS monomers and oligomers, a feature likely responsible for the oligomer-specific binding of PSMα3. Our data further reveal that this region populates a dynamic, yet defined, folded, or partially folded conformation in the oligomer. Importantly, this N-terminal motif remains solvent accessible and targetable in the oligomeric state.

**Figure 5:**
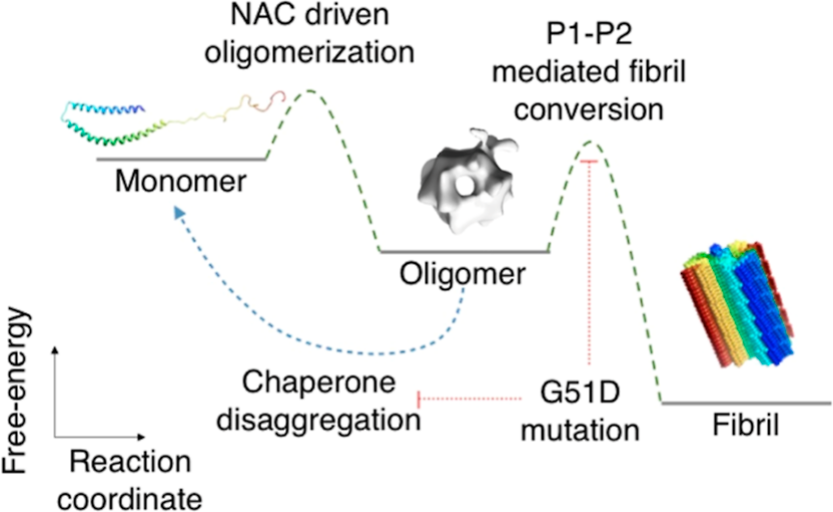
Schematic representation of the αS aggregation landscape.

The PSMα3-mediated inhibition of oligomer-to-fibril conversion prompted us to investigate the role of its binding site, encompassing the P1 and P2 regions, in this essential process of αS pathogenesis (Figure 5). Substantial deletions in both the N-terminal (ΔN11, ΔP1, and ΔP2 variants) and C-terminal (in refs (22 and 36)) regions do not compromise oligomer formation, indicating that the NAC domain acts as the primary driver of oligomerization. Nevertheless, we found that the NAC region alone is insufficient to trigger the conversion of oligomers into fibrils, likely due to its rigidity and burial within the oligomer assembly. Instead, we show that the N-terminal regions that flank the αS NAC domain, involving particularly the P1 and P2 regions therein, modulate oligomer-to-fibril conversion in a sequence-dependent manner, facilitating the escape from the oligomer local thermodynamic minimal toward the fibrillar state.

Considering the oligomer-specific binding of PSMα3, along with its ability to inhibit oligomer-to-fibril conversion and mitigate neurotoxicity,^16^ targeting this specific region emerges as an appealing strategy for designing novel molecular entities mirroring PSMα3 activities. In previous endeavors,^16^ we demonstrated that PSMα3 binding to oligomers is encoded in its physicochemical properties, not contingent on a specific sequence. In this way, sequentially divergent helical peptides with physicochemical traits akin to PSMα3 exhibited comparable inhibitory, binding, and neuroprotective properties.^16^ This knowledge could potentially guide the development of a toolbox of sequentially diverse protein scaffolds mimicking PSMα3 properties to target αS oligomers.^37^

Our findings also provide a molecular framework to rationalize the impact of αS genetic mutations associated with familial Parkinson’s disease on oligomers. Most (but not all) reported familial mutations cluster at P2, having the potential to impact oligomer conformation, fibril conversion, and interaction with other cellular components. Supporting this idea, the G51D amino acid substitution causes a change in oligomer structural dynamics in the N-terminal region, which delays the oligomer-to-fibril transition and presumably results in an accumulation of long-lived, toxic^26^ oligomers that are not efficiently processed by the human disaggregase chaperone network and, instead, capture essential elements of this machinery. This evasion/impairment of proteostasis could explain why this αS mutation triggers the onset of Parkinson’s disease at ages when protein homeostasis is assumed to be preserved.

Finally, it is worth noting that PSMα3 binding reduces oligomer conformational heterogenicity, thereby improving the quality of the 2D image classification in cryoEM, so we could unveil a previously unreported 6-fold symmetry in the oligomer. While limitations in resolution prevented a more detailed structural analysis, this is, to our knowledge, the first proof of a symmetrical supramolecular architecture in αS oligomers. Considering the low molecular weight of PSMα3 (2.6 kDa), it could be expected that larger ligands might exert a greater rigidification effect. Thus, our results encourage the use of P1 and P2 targeting molecules as a new route for advancing oligomer structural characterization.

## Conclusions

Our investigation identifies and characterizes a novel and pathologically relevant region for the implementation of oligomer-targeting strategies while advancing our understanding of oligomer structural properties and the molecular mechanisms that underlie Parkinson’s disease pathogenesis.

## Data Availability

The raw HDX-MS data have been deposited to the ProteomeXchange Consortium via the PRIDE/partner repository with the data set identifier PXD038573. The mass spectrometry proteomics data have been deposited to the ProteomeXchange Consortium via the PRIDE/partner repository with the data set identifier PXD039075. The cryoEM maps for 3DR a-syn C1 and 3DR a-syn:PSMa3 C1 have been deposited at the Electron Microscopy Data Bank with accession codes EMDB-16466 and EMDB-16528, respectively.

## Supporting Information Available

Experimental section, deuterium uptake plots, MAS solid-state NMR analysis, electron microscopy characterization, XL-MS contacts in αS oligomers, SAXS analysis, nsEM micrographs, circular dichroism spectra, migration of the human disaggregase components in the sucrose gradient, sequence coverage, ^13^C chemical shift differences between αS fibrils and oligomers, parameter values obtained from SAXS analysis, cryoEM data acquisition and processing, and HDX data summary (PDF)
